# Genomic features of *Salmonella* Bovismorbificans isolated from calves in British Columbia, Canada

**DOI:** 10.3389/fvets.2025.1590149

**Published:** 2025-06-06

**Authors:** Kazal Ghosh, Melissa Leon, Glenna McGregor, Gitanjali Arya, Stephen Raverty

**Affiliations:** ^1^Animal Health Centre, Government of British Columbia, Abbotsford, BC, Canada; ^2^Western College of Veterinary Medicine, University of Saskatchewan, Saskatoon, SK, Canada; ^3^Public Health Agency of Canada, Guelph, ON, Canada

**Keywords:** *Salmonella* Bovismorbificans, calf, cattle, whole genome sequencing, genomic feature, British Columbia, Canada

## Abstract

*Salmonella enterica* serovar Bovismorbificans has been linked to outbreaks of foodborne human illnesses in the United States and Europe. In mid-2023, *Salmonella* Bovismorbificans was isolated from 4 calves from the Fraser Valley, British Columbia (BC). To our knowledge, this is the first isolation of this pathogen in cattle in BC. The lack of epidemiologic, clinical, and pathologic data concerning *Salmonella* Bovismobificans in British Columbia dairy herds, along with its public health implications, prompted a retrospective review of *Salmonella* isolates recovered at the Animal Health Centre, Abbotsford, BC. We analyzed all *Salmonella* serotypes isolated from cattle between 2008 and 2023. *Salmonella* Dublin and *Salmonella* Typhimurium were the two most frequently isolated serotypes with no isolates of *Salmonella* Bovismorbificans identified between 2008 and mid-2023, and 4 *Salmonella* Bovismorbificans isolations between August and October 2023. These 4 *Salmonella* Bovismorbificans strains (2967, 3266, 3271, and 3876) were subjected to whole genome sequencing. Based on *in-silico* multi-locus sequence typing, the strains were identified as sequence type ST377. Our strains clustered closely with strains recovered from other domestic animals, including cattle, sheep, and goats, from diverse geographical locations, including the USA and Australia. PlasmidFinder software identified the presence of IncFIB and IncFII plasmids in all four strains. A total of 10 SPIs [SPI-1–5, 9, 13–14, centisome 63 (C63PI) and centisome 54 (CS54 island)] were detected in 4 strains except SPI-4 was not observed in strain 2967. A total of 158 virulence genes were predicted across the four strains while one strain (2967) had an additional virulence gene glycosyltransferase operons (*gtrA*) related to immunoinvasion. All four strains carried resistance genes for aminoglycosides, quinolones, peptides, nitroimidazoles, and multi-drug efflux pumps, but no resistance genes were detected for β-lactams, folate pathway antagonists, macrolides, or tetracyclines. Although *Salmonella* Bovismorbificans is not a common serotype in BC dairy herds, the genomic characteristics of the strains highlight the importance of thorough surveillance to monitor potential spread among susceptible herds and animal environments.

## 1 Introduction

*Salmonella* is one of the leading causes of human foodborne infections globally. *Salmonella* comprises two species, six subspecies, and over 2,600 serotypes, all capable of infecting a broad range of animal hosts, including humans (1). In Canada, *Salmonella* is estimated to cause approximately 87,500 cases of human illness, 925 hospitalizations, and 17 deaths annually (2); whereas, in the United States, the annual figures are higher, with approximately 1.35 million infections, 26,500 hospitalizations, and 420 deaths reported yearly (3). Among the various *Salmonella* serotypes, *Salmonella* Typhimurium and *Salmonella* Enteritidis are most commonly associated with human foodborne infections, while *Salmonella* Bovismorbificans is less frequently recovered from humans (4, 5). However, outbreaks of *Salmonella* Bovismorbificans in people have been documented in several countries and linked to various food sources, including contaminated pork products, tahini, hummus, salad products and sprouted seeds (6, 7).

In the dairy industry, salmonellosis is a significant cause of morbidity and mortality in calves and cows, often manifesting as individual cases of diarrhea or herd outbreaks. Common serotypes associated with clinical disease include *Salmonella* Typhimurium, Dublin, Anatum, and Montevideo (8, 9). More recently, *Salmonella* Bovismorbificans has emerged as a potentially significant pathogen of dairy cattle in the United States and New Zealand (6, 10). Infections often result in considerable economic loss due to high morbidity, costs of medication, reduced fecundity, milk yields, and growth, which may be further exacerbated by rapid spread of the pathogen. Clinical signs include fetid and occasionally hemorrhagic diarrhea, abortion and septicemia. *Salmonella* can readily be transmitted through contaminated environments, fomites, feed, and water.

The lack of information on the natural history of *Salmonella* Bovismorbificans, presents unique challenges in understanding its pathogenicity, epidemiology, environmental or enteric persistence, intervention and control. While virulence factors of this bacterium remain underexplored, recent molecular advancements have provided insights into its genomic characteristics. Sequence type ST377, and ST142, appeared to be the predominant *Salmonella* Bovismorbificans in foodborne illnesses in the USA and Europe (6, 11). Antimicrobial resistance, including multidrug resistance, has been observed in this serotype in several studies and plasmid-encoded antimicrobial resistance genes, *bla*_*DHA*−1_ and *qnrB4*, were identified in two *Salmonella* Bovismorbificans isolates in a recent study (12–14). Additionally, a human isolated multidrug-resistant *Salmonella* Bovismorbificans harbored the *bla*_*SHV*5_-type extended-spectrum β-lactamase gene, marking the first report of such resistance in this serotype (15). *Salmonella spp*. require multiple genes for full virulence, many of which are located in pathogenicity islands (SPIs) on the chromosome. A total of 21 SPIs have been identified in *Salmonella* spp. (16), with *Salmonella* Typhimurium containing at least five SPIs that confer specific virulence traits and may be acquired through horizontal gene transfer (17). In *Salmonella* Bovismorbificans, there are several SPIs (1, 2, 4, 5, 9, and 11) which are largely synonymous to the genome of *Salmonella* Typhimurium LT2 (18). Genetic analysis of *Salmonella* Bovismorbificans strains identified several pathogenicity island genes, including *avrA, ssaQ, mgtC, spi4*, and *sopB*, but lacked certain phage-related genes (15). One previous study in Hungary suggests that while *Salmonella* Bovismorbificans is less invasive than other *Salmonella* serotypes, it can still colonize and persist in the gastrointestinal tract, posing a contamination risk for meat products (15).

Based on review of the Animal Health Centre laboratory database, to the best of our knowledge, no isolates of *Salmonella* Bovismorbificans were recorded in British Columbia cattle prior to mid-2023. Since the initial isolation in 2023, *Salmonella* Bovismorbificans has been recovered from 4 dairy calves that presented with diarrhea, or septicemic salmonellosis/acute death with no premonitory signs. Several *Salmonella* serotypes pose a major risk to the dairy industry primarily due to gastrointestinal illness in calves. For this reason, the ability to rapidly distinguish serovars by an advanced understanding of the genomic characteristics of this pathogen is essential to assess its pathogenicity and epidemiology. Further understanding of these aspects of the bacterium will ultimately contribute to development of effective disease control and management strategies. Investigating the possible persistence of this pathogen in the environment and host animals may provide additional insight into the transmission of *Salmonella* Bovismorbificans which is critical for devising strategies to reduce infection risks in animals and exposure to humans. To the best of our knowledge, there has been a lack of information on the genomic features of *Salmonella* Bovismorbificans isolates from calves. The goal of this study is to examine and describe the genomic characteristics of *Salmonella* Bovismorbificans isolated from 4 calves presenting with diarrhea (3 calves), or septicemic salmonellosis (1 calf).

## 2 Methodology

### 2.1 Sample description

The Animal Health Centre (AHC, Abbotsford, British Columbia) is the provincial veterinary diagnostic laboratory for British Columbia that receives a wide array of samples from production, companion, wild, and exotic animals. There has been an ongoing effort to survey for *Salmonella* from intestine and fecal samples by selective culture. Fecal or tissue samples (small and large intestine) were initially enriched in selenite broth at 42°C for 24 h, then streaked onto Hektoen and XLT4 agars (Oxoid, Ontario, Canada) and incubated aerobically at 35°C for 24 to 48 h. MALDI-ToF MS (Bruker, Ontario, Canada) and basic biochemical tests (Gram staining, Oxidase, and Indole tests) were then performed to identify typical *Salmonella* colonies. *Salmonella* serogroups were determined by slide agglutination testing, and *Salmonella*-positive isolates forwarded to the Division of Enteric Diseases of the National Microbiology Laboratory, Public Health Agency of Canada (PHAC) in Guelph, Ontario, for serotype confirmation using whole genome sequencing through the Salmonella *In Silico* Typing Resource (SISTR) (19). As *Salmonella* Bovismorbificans was isolated for the first time in calves at our laboratory in the year 2023 and limited information was available regarding its genetic features, we obtained the raw genome sequences from PHAC in fastQ file format for four *Salmonella* Bovismorbificans strains (2967, 3266, 3271, and 3876, respectively). Strain 3876 was isolated from the colon of a calf with septicemia, whereas the other strains were obtained from the feces of calves with diarrhea.

### 2.2 DNA library preparation and whole genome sequencing

Genomic DNA was extracted from pure Salmonella cultures using the LuminUltra RNA 1K 480 commercial assay with the Thermofisher Kingfisher Flex (VWR) platform. The protocol from this kit was modified and verified for doing bacterial DNA extractions. Modifications included using 1 ml of overnight bacterial broth culture (in place of patient sample), doubling the volume of magnetic beads (to increase yield), increasing the time of lysis step from 10 min to 1 h along with the addition of proteinase K (Applied Biosciences) and heat during lysis. Extracted DNA was quantified using the FilterMax Multimode Reader F5 (Molecular Devices) and the quant-iT dsDNA assay kit (Invitrogen) and diluted down to a genomic DNA concentration of 0.2 ng/μl. Sample libraries for all isolates were prepared using the Illumina Nextera XT library preparation kit (Illumina, Inc., San Diego, CA, United States). Paired end sequencing was performed either on the Illumina NextSeq 550 using the V2.5 mid output 300 cycle kit (2 × 150 reads) or on the NextSeq 1000 using the P1 Reagent kit, also 300 cycles (2 × 150 reads) to achieve a minimum coverage of equal to or >40 × for all strains.

### 2.3 Genome assembly and annotation

All raw sequencing reads were quality-checked using FastQC (v0.12.1) and trimmed with Trimmomatic (v0.39). The trimmed reads from all four strains were then *de novo* assembled using Unicycler (v0.5.0). The quality of the draft genome assemblies was assessed using QUAST (v5.2.0) and genome completeness of the four strains was evaluated by BUSCO (v5.6.1). The average nucleotide identity (ANI) was calculated by comparing the assembled genomes of our studied strains with *Salmonella* Bovismorbificans strain CVM 30176 (GenBank accession number CP051349.1) using the ANI calculator (20). The assembled genomes were initially annotated using the Rapid Annotation using Subsystem Technology (RAST) server version 2.0 (21).

### 2.4 Pan-genome, single-nucleotide polymorphism (SNP) phylogeny and multi-locus sequence type analysis

For SNP analysis, a dataset was created comprising our four studied strains and a total of 110 *Salmonella* Bovismorbificans genomes downloaded from NCBI GenBank. The genome accession numbers, and their corresponding metadata are provided in Supplementary Table 1. Sequence types (STs) for all 114 genomes were determined using the MLST database (v2.0) at the Center for Genomic Epidemiology (CGE; https://cge.food.dtu.dk/services/MLST/) and Pangenome analysis was performed using Roary (v3.13.0) on the GFF files generated by Prokka (v1.14.6). A minimum 95% identity for blastp and a core gene requirement of 99% for isolates were selected. MAFFT (v7.520) was used as part of the Roary pipeline to create a core genome alignment. This core genome alignment was used as input for SNP identification with SNP-sites (v2.5.1). The resulting core genome SNP alignment was then used to construct a maximum parsimony phylogenetic tree with the program RAxML (v1.2.1), using the general time reversible (GTR) model of nucleotide substitution and the Gamma model of rate heterogeneity. The tree was visualized with iTOL (22) and annotated with STs, host information, and country of origin to compare genetic diversity.

### 2.5 Salmonella pathogenicity islands (SPIs), plasmid, virulence genes and antimicrobial resistant gene prediction

The assembled genomes of our studied stains were analyzed using the CGE SPIFinder (v2.0) tool (https://cge.food.dtu.dk/services/SPIFinder/) to identify *Salmonella* pathogenicity islands (SPIs). The analysis was performed using the default settings of SPIFinder 1.0, with a 95% identity threshold and a minimum length of 60%. PlasmidFinder (v2.1; https://cge.food.dtu.dk/services/PlasmidFinder/) was used to identify plasmids, with a minimum identity of 95% and minimum coverage of 80%, which were further confirmed using NCBI Nucleotide BLAST. Antibiotic-resistant genes were identified and confirmed among the isolates using the Resfinder (v4.6.0) (23) and CARD database (24). To predict the occurrence of various virulence determinants listed in the Virulence Factor Database (VFDB) among our studied strains VFanalyzer (25) was used. Additionally, we randomly selected 10 *Salmonella* Bovismorbificans strains from our dataset and obtained additional 13 different *Salmonella* serotypes (Supplementary Table 1) listed in VFanalyzer to screen for virulence genes for comparative pathogenomics (25). A heatmap was constructed to show the presence or absence of selected virulence genes across all the strains, using the pheatmap package in RStudio (v1.1.456). For all software used, default parameters were applied unless otherwise specified.

### 2.6 Phenotypic resistance testing

Phenotypic resistances of the four *Salmonella* Bovismorbificans strains from our study were determined using the Kirby-Bauer disk diffusion assay (26) against the following antibiotics: Ampicillin (10 μg), Ceftiofur (30 μg), Erythromycin (15 μg), Enrofloxacin (5 μg), Gentamicin (10 μg), Penicillin (10 μg), Tetracycline (30 μg), and Sulfamethoxazole/Trimethoprim (25 μg). The size of the zone of inhibition was interpreted according to the Clinical and Laboratory Standards Institute (CLSI) guidelines (27).

## 3 Results

### 3.1 Salmonella serotypes identified at the Animal Health Centre and general genomic features of *Salmonella* bovismorbificans

We analyzed all *Salmonella* serotypes isolated from cattle at the AHC between 2008 and 2023. A total of 12 different serotypes were identified during this period, with *Salmonella* Dublin and *Salmonella* Typhimurium being the most prevalent (Table 1). However, *Salmonella* Bovismorbificans was isolated for the first time in mid-2023, and since then, we have observed this organism in three 10 to 14-day-old calves with diarrhea (strains 2967, 3266, and 3271) from one farm and one 14-day-old calf with septicemic salmonellosis (strain 3876) from another farm. The assembled genomes of *Salmonella* Bovismorbificans strains 2697, 3266, 3271, and 3876 were 4,806,605 bp, 4,814,212 bp, 4,818,110 bp, and 4,815,289 bp long, respectively, and composed of 39, 27, 27, and 33 contigs. The GC content for all four strains was 52.2% (Table 2). BUSCO estimated genome completeness to be 98.4% for each of the strains in this study. The average nucleotide identity (ANI) of these strains was 99.9% when compared to *Salmonella* Bovismorbificans strain CVM 30176. Initial annotation results from RAST server predicted 4,511, 4,546, 4,546, and 4,547 coding sequences (CDS) for strains 2697, 3266, 3271, and 3876, respectively. All isolates contained 77 tRNA and 1 tmRNA.

**Table 1:** Number of different Salmonella serotypes isolated from cattle at Animal Health Centre, British Columbia from 2008 to 2023.

**Salmonella serogroup**	***Salmonella* serotype name**	**Total number of isolates**
B	*Salmonella* Typhimurium	73
B	*Salmonella* Heidelberg	6
B	*Salmonella* Schwarzengrund	2
C1/C4	*Salmonella* Infantis	1
C2/C3	*Salmonella* Newport	1
C2/C3	***Salmonella*** Bovismorbificans^*^	4
D1	*Salmonella* Dublin	135
D1	*Salmonella* Enteritidis	7
D1	*Salmonella* I:ROUGH-O:g,p:-	4
D1	*Salmonella* I:9,12:-:-	2
E123	*Salmonella* Uganda	2
G1/G2	*Salmonella* Worthington	2

^*^Isolated in the year 2023.

**Table 2:** Summary of genome assembly and annotation for four *Salmonella* Bovismorbificans strains.

**Strains**	**2967**	**3266**	**3271**	**3876**
**Alignment-based statistics**				
Total length	4,806,605	4,814,212	4,818,110	4,815,289
Number of contigs	39	27	27	33
Largest contig	828,803	868,677	1,247,819	738,958
N50	373,615	431,721	421,952	431,820
GC (%)	52.22	52.22	52.21	52.21
Genome completeness	98.4%	98.4%	98.4%	98.4%
Average nucleotide identity (ANI)	99.4%	99.9%	99.9%	99.9%
**Annotation findings**				
CDS	4,511	4,546	4,546	4,547
rRNA	1	2	2	2
tRNA	77	77	77	77
tmRNA	1	1	1	1

### 3.2 Pan-genome, SNP phylogeny and MLST

Pan-genome analysis of our four studied strains, along with 110 *Salmonella* Bovismorbificans genomes from NCBI GenBank, revealed a total of 9,624 genes. Of these, 3,757 were core genes (present in >99% of isolates), 50 were soft core genes (present in 95–99% of isolates), shell genes were present in 15–95% of isolates, and cloud genes were present in 0–15% of isolates. The pan-genome details, including the gene absence/presence table, are in Supplementary Table 1.

To investigate the genetic diversity of our four studied strains, 110 *Salmonella* Bovismorbificans genomes from a variety of hosts, including cattle, humans, sheep, goats, swine, horses, and environmental sources, were obtained from NCBI GenBank (accessed on January 10, 2024). These genomes were from disparate geographic locations, including Australia, the USA, Switzerland, and the United Kingdom. Sequence type (ST) was determined using the MLST database. A total of five different STs (377, 1499, 142, 150, and 2640) were identified, with all four strains from this study typed as ST377.

**Figure 1:**
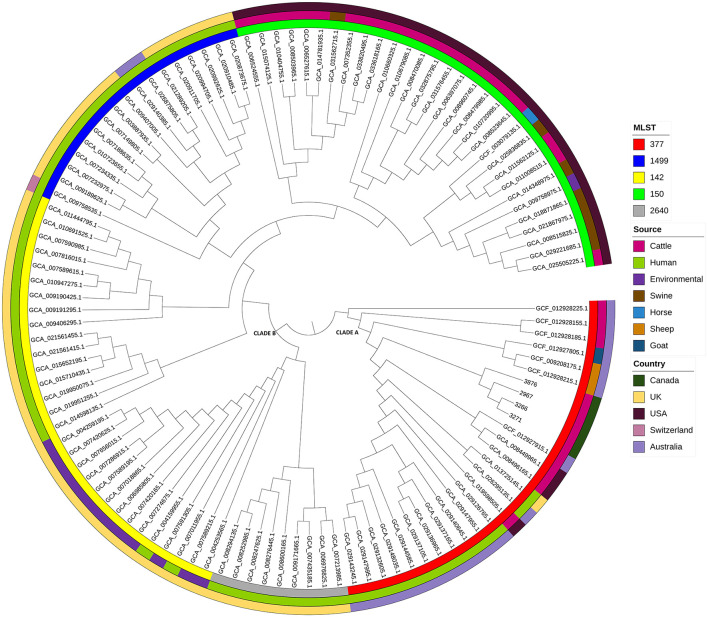
Phylogenetic tree of the four *Salmonella* Bovismorbificans strains analyzed in this study, along with 110 strains obtained from NCBI GenBank, based on core genome SNPs. This tree is annotated with Multi-Locus Sequence Typing (MLST), isolate source, and country of origin. The tree was constructed using maximum parsimony analysis with RAxML and visualized using iTOL.

A phylogenetic tree was constructed based on an SNP-based core-genome alignment, and the tree was annotated with STs, isolation sources, and countries (Figure 1). Two major clades were identified. In clade A, all our studied strains grouped with other ST377 strains, while in clade B, the remaining STs were clustered together. Within clade A, our strains clustered closely together with isolates recovered from other domestic animals, including cattle, sheep, and goats, from different geographical locations, including the USA and Australia.

### 3.3 Salmonella pathogenicity islands (SPIs), plasmid and virulence associated genes

**Figure 2:**
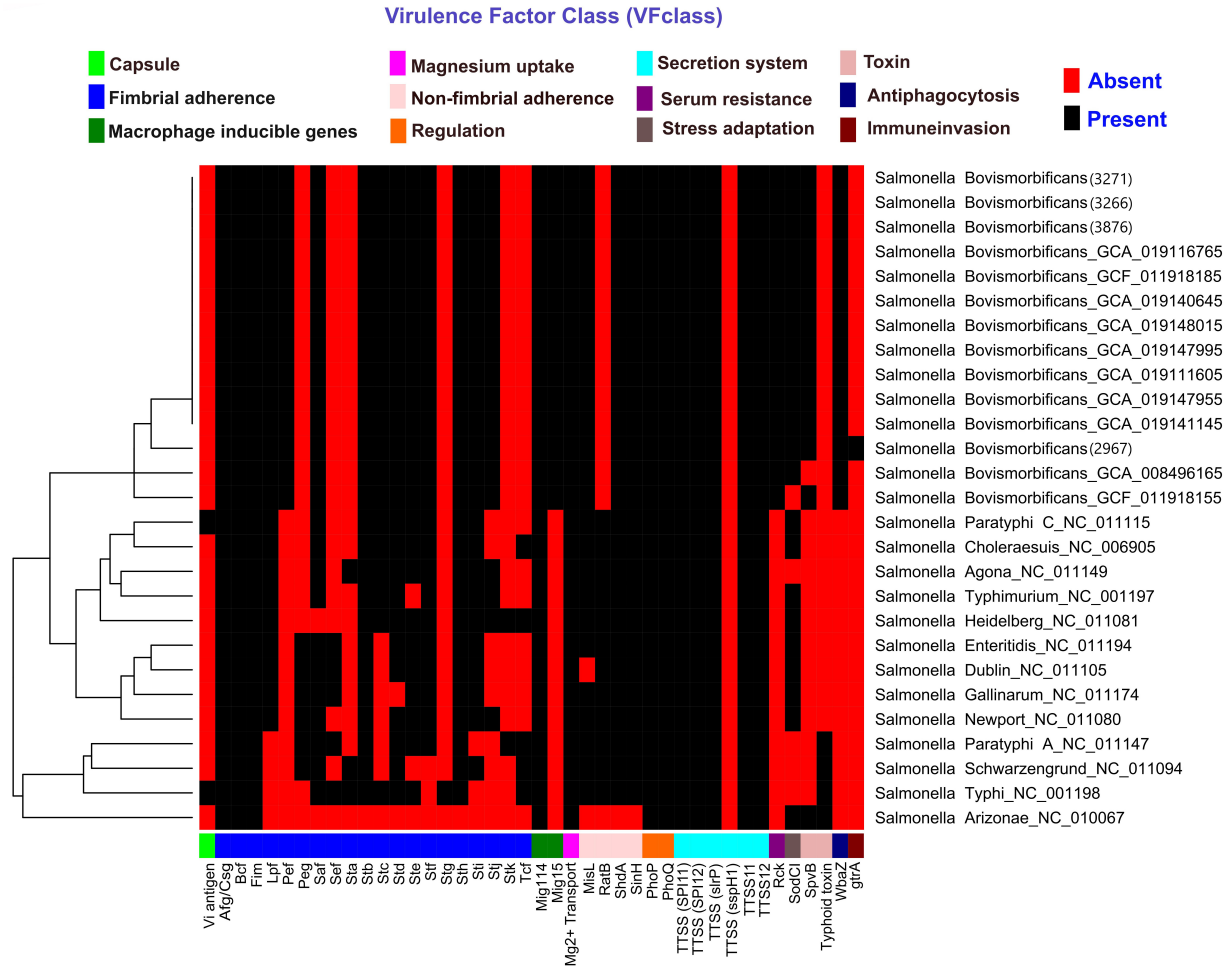
Heatmap displaying the distribution of virulence factors and associated genes in BC strains, 10 randomly selected Salmonella Bovismorbificans isolates, and 13 different Salmonella serotypes. The heatmap was generated using the pheatmap package in RStudio (v1.1.456). Red indicates the absence of a gene, while black signifies its presence.

Plasmid finder determined that our studied strains contained IncFIB and IncFII plasmids. A total of 10 *Salmonella* pathogenicity islands (SPIs)—specifically SPI-1 to SPI-5, SPI-9, SPI-13 to SPI-14, centisome 63 (S73PI), and centisome 54 (CS54 island)—were detected across the four strains examined. Notably, SPI-4 was absent in strain 2967. All the SPIs details are in Supplementary Table 2. Virulence gene prediction was performed on our studied strains, including 10 randomly selected *Salmonella* Bovismorbificans strains from the generated dataset, as well as 13 different *Salmonella* serotypes from VFanalyzer. A total of 158 virulence genes were predicted across our four strains, belonging to 10 different virulence classes, including: fimbrial adherence determinants, non-fimbrial adherence determinants, macrophage-inducible genes, magnesium-uptake genes, regulation, serum resistance, stress adaptation, toxins, anti-phagocytosis, and secretion system genes. An additional virulence gene, *gtrA* (glycosyltransferase operons, related to immune invasion), was predicted only in strain 2967. All of our strains harbored several genes related to the type III secretion system (T3SS) encoded in *Salmonella* pathogenicity islands 1 (SPI-1) and 2 (SPI-2). However, no strains contained any capsule-related genes, which were only found in *Salmonella* Typhi and Paratyphi. A heatmap was generated for all 14 *Salmonella* Bovismorbificans strains, including virulence genes from 13 different *Salmonella* serotypes (Figure 2). Our studied strains clustered together with other *Salmonella* Bovismorbificans strains based on the presence or absence of virulence genes. A list of all detected genes is provided in Supplementary Table 1.

### 3.4 Resistant profile and antimicrobial resistant genes (ARGs)

Strains 2967 and 3876 were phenotypically resistant to Ampicillin, Enrofloxacin, and Penicillin, while strain 3266 and strain 3271 were resistant to Enrofloxacin, Penicillin, and Erythromycin, Penicillin, respectively (Table 3). The Resfinder and CARD database was used to predict the antimicrobial resistance genes (ARGs) in these four studied strains. A total of 29 antimicrobial resistance genes from various classes were identified in strains 3271 and 2967, while 30 antimicrobial resistance genes were detected in strains 3266 and 3876. However, only ARGs detected based on a protein homolog model with more than 90% identity to reference genes were included in this study. All four isolates harbored similar resistance genes associated with aminoglycosides, quinolones, peptides, nitroimidazoles, and multi-drug efflux pumps. No ARGs were detected for β-lactams, folate pathway antagonists, macrolides, or tetracyclines.

**Table 3:** Summary of phenotypic antimicrobial susceptibility test results and list of antimicrobial resistant genes detected by Resfinder and CARD database.

**Strains**	**Phenotypic resistance**	**Antimicrobial resistance genes**								
		**Aminoglycoside**	β**-lactams**	**Quinolone**	**Folate pathway antagonist**	**Peptide**	**Macrolide**	**Tetracycline**	**Nitromidazole**	**Multi-drug efflux pump**
2967	AMP, ENRO, PEN	*AAC(6′)-Iy, kdpE*	-	*emrB, emrR, MdtK*	-	*bacA*	-	-	*msbA*	*golS, sdiA, CRP, marA, H-NS, acrA, acrB*,
3266	ENRO, PEN	*AAC(6′)-Iy, kdpE*	-	*MdtK, emrB, emrR*	-	*bacA*	-	-	*msbA*	*golS, MdtK, sdiA, CRP, marA, H-NS, acrA,acrB*
3271	ERY, PEN	*AAC(6′)-Iy, kdpE*	-	*MdtK, emrB, emrR*	-	*bacA*	-	-	*msbA*	*golS, MdtK, sdiA, CRP, marA, H-NS, acrA,acrB*
3876	AMP, ENRO, PEN	*AAC(6′)-Iy, kdpE*	-	*MdtK, emrB, emrR*	-	*bacA*	-	-	*msbA*	*golS, MdtK, sdiA, CRP, marA, H-NS, acrA,acrB*

EMRO, Enrofloxacin; AMP, Ampicillin; PEN, Penicillin; ERY, Erythromycin.

## 4 Discussion

Diarrhea is a leading cause of dairy calf mortality and enteric *Salmonella enterica* infections, especially *Salmonella* Typhimurium, has been associated with an increased risk of morbidity and mortality (28). *Salmonella enterica* infections have been associated with severe intestinal lesions, including the presence of fibrin in the feces, and fatal septicemia (28). Although *Salmonella* Bovismorbificans has been identified in various foodborne sources, worldwide reports of this bacterium from dairy calves are limited. Recent studies suggest this serotype is an emerging pathogen in dairy farms; this bacterium has been reported in dairy environments in the USA and in New Zealand (6, 7). Since 2015 in New Zealand, *Salmonella* Bovismorbificans has become more frequently detected in adult dairy cows and calves on dairy farms or calf-rearing operations (7). Due to the persistence of *Salmonella* Bovismorbificans in the environment, transmission from cattle to humans and other susceptible animals has been proposed. In Scotland, *Salmonella* Bovismorbificans recovered from gray seals closely matched isolates from cattle, indicating land-sea pathogen transfer (29).

In dairy cattle, *Salmonella* Dublin and *Salmonella* Typhimurium are the most prevalent serotypes worldwide, including the USA (30). Our findings align with this, as these two serotypes were the most frequently identified in our laboratory (Table 1). However, to our knowledge, no prior publications describe isolation of *Salmonella* Bovismorbificans from calves in Canada. Food and human isolates of *Salmonella* Bovismorbificans in Europe have been associated with ST142 (31). ST142 and ST377 are the predominant sequence types associated with foodborne illnesses (6). Downloaded *Salmonella* Bovismorbificans ST377 strains from NCBI GenBank showed that this sequence type has been isolated primarily from ruminants (cattle, sheep, goat) and humans. Our studied strains are homologous to ST377 and clustered with *Salmonella* Bovismorbificans isolated from cattle, sheep and goat from Australia and the USA. A sub-cluster strain of *Salmonella* Bovismorbificans ST377 has also been associated with a hummus sourced human outbreak and closely related to *Salmonella* Typhimurium and *Salmonella* Muenchen (6). Our strains clustered in Clade A with other strains of ST377, whereas other sequence types (ST1499, 142, 150, 2640) were clustered together in Clade B. In contrast, a whole genome core genes study assigned ST150 to its own cluster based on significant differences from the other sequence types, including ST377, which were similar and clustered together phylogenetically (6).

Many mechanisms that are involved in bacterial cellular invasion, survival, and replication inside phagocytic cells are encoded by genes in the *Salmonella* pathogenicity islands (SPIs) (8). A total of 21 SPIs had been identified in *Salmonella* spp. (16). Total 10 different SPIs were determined in our studied strains (SPI-1-5, 9, 13, 14, C63PI and CS54 island). In *Salmonella* Bovismorbificans, SPI-1, 2, 4, 5, 9 and 11 were largely identical to the genome of *Salmonella* Typhimurium LT2, while SPI-3, 6, 10 and 12 featured deletions (18). Other studies had not detected SPI-13, 14, 15, and 17 in *Salmonella* Bovismorbificans (18). SPI-1-5 are the major SPIs where virulence factors are encoded (16). Similarly, a study of 110 Hungarian strains of *Salmonella* Bovismorbificans demonstrated virulence genes *avrA, ssaQ, mgtC, spi4*, and *sopB*, which were located on SPI-1 to 5, respectively (15). All of our strains contained SPI-1 and−2, with T3SS associated with each island. SPI-1 and−2 each encode their own T3SS to translocate effectors to the host cell (16). SPI-1 mediates invasion, which activates SPI-2 to replicate inside host cells (16) and SPI-1 and SPI-3 contribute to replication in macrophages. Both these genes are regulated by the PhoP-PhoQ system (16). SPI-4 contributes to colonization of the intestine in cattle and encodes a type 1 secretion system (T1SS) (16). In bovine ligated intestinal loops, SPI-5 evokes enterocolitis with *Salmonella* Dublin challenge (16). Unlike *Salmonella* Typhimurium LT2, the SPI-6 in a human *Salmonella* Bovismorbificans isolate was diminished and retains only a portion of the fimbrial *saf* operon (18). This isolate lacked the Type VI secretion system (T6SS), which was similar to other *Salmonella* spp. Serotypes (18).

As IncFIB and IncFII plasmids may have both antimicrobial resistance genes and various virulence factors, their spread in foodborne pathogens is a significant public health concern. These plasmids are frequently found in *Salmonella* spp. and avian pathogenic *E. coli* which may be reservoirs, facilitating plasmid transfer to other Gram-negative bacteria (32, 33). Similar to other published case series, all of our strains had both IncFIB and IncFII plasmids.

As with other *Salmonella* serotypes, *Salmonella* Bovismorbificans has an array of virulence factors that enable host colonization, invasion, and survival. These virulence factors have crucial roles in the pathogenesis of infection, that range from initial attachment to host cells to evasion of the immune response. While the specific virulence factors of *Salmonella* Bovismorbificans have not yet been as extensively characterized as those of other *Salmonella* serotypes, the general mechanisms underlying *Salmonella* pathogenesis may be extrapolated to understand its virulence. Based on virulence profile, results from our *Salmonella* Bovismorbificans strains clustered together, but distinct to other *Salmonella* serovars. Three of our strains (3266, 3271, and 3876) had similar virulence genes, while one strains (2967) had one additional virulence gene *gtrA* that may be responsible for immune invasion. Our strains had T3SS encoded in several *Salmonella* pathogenicity islands (SPI-1 and SPI-2), as well as multiple genes responsible for fimbrial and non-fimbrial adherence, macrophage inducible genes and genes responsible for stress adaptation. In one study, all of the human *Salmonella* Bovismorbificans isolates in Malawi carried a virulence plasmid (pVIRBov), which was variably detected in *Salmonella* isolates from animals (18). This plasmid is similar to the *Salmonella* Typhimurium LT2 virulence plasmid pSLT, which carries the *spv* virulence gene cassette and the *pef* (plasmid-encoded fimbriae) operon that mediates adhesion to intestinal epithelial cells (18). In our strains, several genes responsible for fimbrial adherence, including *pef* , were found. The gene *pefA* has been detected in *Salmonella* Typhimurium and is associated with diarrhea, enteritis and fibrinosuppurative splenitis (8). In *Salmonella* Typhimurium, *lpfC* and *pefA* genes mediate adhesion to the intestinal cells, then *lpfC* and bovine colonization factor (*Bcf* ) facilitate invasion of Peyer's patches for long-term intestinal persistence (8). These *lpf* and *Bcf* genes were present in all our strains. Downstream from the *pef* operon on pVIRBov was the *rcK* (resistance to complement killing) gene, which is also found in *Salmonella* Typhimurium. This *rcK* gene was found in all our strains. This gene confers resistance to complement-mediated bactericidal activity, prevents the membrane attack complexes from forming fully and is associated with enhanced bacterial survival in macrophages and virulence (16, 18). Moreover, all the strains from our case series and in a prior report of a human derived isolate of *Salmonella* Bovismorbificans, the following fimbrial operons were found: *stf, saf, stb, fim, stc, std, lpf, stj, sth, bcf, sti, csg* and *pef* . These operons have also been identified in *Salmonella* Typhimurium (18). Fimbriae mediates the initial attachment of the bacteria to intestinal epithelial cells and is accompanied by the T3SS to invade epithelial cells (16). Two non-fimbrial intestinal colonization factors (*MisL* and *ShdA*) were also detected in our isolates. These colonization factors have a predilection for Peyer's patches of the terminal ileum for efficient invasion and replication and have been associated with prolonged fecal shedding of *Salmonella* Typhimurium (16). *Salmonella* plasmid virulence (*spv*) genes are required for pathogenicity and were localized on large plasmids (34). A 90 kbp plasmid was identified in multiple *Salmonella* Bovismorbificans strains with a 3–5 kbp *Hin* dIII fragment, which was homologous to the *spvBC* genes of *Salmonella* Typhimurium (34). Eighty percent (88 out of 110) of isolates from Hungary had the *spvC* gene, which was found on a virulence plasmid (15). Although our strains did not have the *spvBC* or *spvC* genes, *spvB* was detected and has been associated previously with intracellular growth within the host and inhibiting autophagy (8). The proteins that encode *spvB* are translocated to the cell by T3SS of SPI-2 (8). This gene was found on a very transmissible plasmid in *Salmonella sp* (8). All four of our strains, and all 110 Hungarian strains of *Salmonella* Bovismorbificans, had the virulence gene *sodC1*, which is a phage-related gene (15).

Phenotypically all four bacterial strains were resistant to penicillin, three of them (2967, 3266 and 3876) were resistant to enrofloxacin, with two of them (2967 and 3876) resistant to ampicillin and one (3271) resistant to erythromycin. All four strains had similar antimicrobial resistant genes for aminoglycosides, quinolone, peptide, nitroimidazole and multi-drug efflux pump. The Canadian Integrated Program for Antimicrobial Resistance Surveillance (CIPARS) has recently reported an increase in extended-spectrum beta-lactamase (ESBL)-producing *Salmonella* isolated from humans, animals, and food in Canada (35). An ESBL-producing *Salmonella* Bovismorbificans with a CTX-M-9 enzyme (gene *bla*_CTX − M−9_) was isolated in a human in Portugal (31) and a *bla*_SHV5_-type ESBL gene was detected in another human isolate from Hungary (15). Two *Salmonella* Bovismorbificans isolated from food products in China also produced ESBL and contained the genes *bla*_OXA − 1_ and *bla*_DHA − 1_ (13). Although our strains showed phenotypic resistance to β-lactams, we did not detect any genes responsible for β-lactamases. Another study on human *Salmonella* Bovismorbificans strains had a number of putative β-lactamase genes that showed phenotypic resistance to cephalosporins (cefuroxime), but were susceptible to ampicillin (18). No strains from our study were resistant to tetracycline. Ironically, in New Zealand, between 2018 and 2023, there was an increasing trend of *Salmonella* Bovismorbificans isolates resistant to tetracycline, however, a putative downward trend has been inferred by declining oxytetracycline sales subsequent to 2020 (7). Tetracycline resistant *Salmonella* Bovismorbificans with the tetracycline resistance gene *tet(A)* were isolated from food products in China (13). In our case series, no strains were resistant to sulfamethoxazole or trimethoprim. In Thailand, most *Salmonella* cases including *Salmonella* Bovismorbificans were isolated from goats and susceptible to all tested antimicrobials except for sulfamethoxazole. This anomaly may be attributed to the widespread use of sulfamethoxazole as an anti-diarrhetic in goats in Thailand (36). Eleven of 14 human isolates of *Salmonella* Bovismorbificans from Malawi had phenotypic resistance to sulfamethoxazole (18). Although there were no genes associated with resistance to sulfamethoxazole, a gene associated with resistance to trimethoprim (*dhfr1*) was detected in these Malawian isolates (18). Similarly, two *Salmonella* Bovismorbificans isolates from food products in China were phenotypically resistant to sulfamethoxazole and trimethoprim, however, had a plasmid that contained the sulphonamide resistance genes *sul1* and *sul2* (13). Antimicrobial resistance genes to quinolone (*emrB, emrR, Mdtk*) were detected in all four of our strains; enrofloxacin resistance phenotypes were identified in three of these strains. Quinolone resistance is mediated by the target protection mechanisms encoded by *qnr* genes (37). In China, transferrable plasmids with genes *qnrD* and *qnrB4* were reported in *Salmonella* Bovismorbificans recovered from human and food products (13, 37). In addition, the aminoglycoside resistance gene *acc(6*′*)Ib-cr* may also encode for enzymatic modifications toward quinolone antimicrobials, ciprofloxacin and norfloxacin (37). The plasmids containing gene *qnrB4* also contained aminoglycoside resistance genes *aac(6*′*)-Ib-cr* and *aac(**3**)-IV* (13). In all four of our strains, the aminoglycoside resistance gene *aac(6*′*)-Iy* was detected by Resfinder.

Outbreaks of *Salmonella* are commonly observed after flooding, especially when cattle feed and equipment are contaminated with floodwaters carrying the bacteria. Severe flooding in Fraser Valley, British Columbia, in November 2021 may have introduced this serotype into the local cattle population. A notable epidemic of *Salmonella* Bovismorbificans was reported in 1978 on a New Zealand dairy farm, where a broken water pipe created a muddy pond in the paddock (38). During this outbreak, 20 cows died from salmonellosis, 10 calves were either stillborn or died shortly after birth, and 10 cows were culled due to poor response to treatment. Clinical cases began 5 days after the cattle were removed from the paddock, and *Salmonella* Bovismorbificans was still present in fecal samples and in the soil up to 6 months later (38).

## 5 Conclusions

The genomic characteristics of the BC dairy isolates suggest that *Salmonella* Bovismorbificans is a putative pathogen associated with diarrhea in calves. There is little information on the epidemiology and pathogenicity of *Salmonella* Bovismorbificans in Canadian dairy herds. A limitation of our study was the use of isolates obtained through our laboratory submissions only; however, current findings provide baseline molecular information for prospective investigations. Given the potential risk of food borne transmission and genomic features of the serotype observed in our study, we recommend continued monitoring for this pathogen to detect, manage and mitigate any future outbreaks.

## Data Availability

All the details of the genomic analysis are in Supplementary material. We also submitted assembled genome of all four studied strains in NCBI GenBank (BioProject ID: PRJNA1233353). For further inquiries, please contact the corresponding author.

## Supplementary material

Supplementary Table 1 Details of the dataset, pan-genome analysis, and identified virulence genes across the studied isolates.

Supplementary Table 2 Details of the Salmonella pathogenicity islands (SPIs) present in the four studied BC strains.
